# Variation in photosynthesis and stomatal conductance among red maple (*Acer rubrum*) urban planted cultivars and wildtype trees in the southeastern United States

**DOI:** 10.1371/journal.pone.0197866

**Published:** 2018-05-24

**Authors:** Eleanor C. Lahr, Robert R. Dunn, Steven D. Frank

**Affiliations:** 1 Department of Entomology and Plant Pathology, North Carolina State University, Raleigh, North Carolina, United States of America; 2 Department of Applied Ecology, North Carolina State University, Raleigh, North Carolina, United States of America; Eidgenossische Forschungsanstalt fur Wald Schnee und Landschaft Institut fur Schnee- und Lawinenforschung, SWITZERLAND

## Abstract

Photosynthesis is a fundamental process that trees perform over fluctuating environmental conditions. This study of red maple (*Acer rubrum* L.) characterizes photosynthesis, stomatal conductance, and water use efficiency in planted cultivars relative to wildtype trees. Red maple is common in cities, yet there is little understanding of how physiological processes affect the long-term growth, condition, and ecosystem services provided by urban trees. In the first year of our study, we measured leaf-level gas exchange and performed short-term temperature curves on urban planted cultivars and on suburban and rural wildtype trees. In the second year, we compared urban planted cultivars and urban wildtype trees. In the first year, urban planted trees had higher maximum rates of photosynthesis and higher overall rates of photosynthesis and stomatal conductance throughout the summer, relative to suburban or rural wildtype trees. Urban planted trees again had higher maximum rates of photosynthesis in the second year. However, urban wildtype trees had higher water use efficiency as air temperatures increased and similar overall rates of photosynthesis, relative to cultivars, in mid and late summer. Our results show that physiological differences between cultivars and wildtype trees may relate to differences in their genetic background and their responses to local environmental conditions, contingent on the identity of the horticultural variety. Overall, our results suggest that wildtype trees should be considered for some urban locations, and our study is valuable in demonstrating how site type and tree type can inform tree planting strategies and improve long-term urban forest sustainability.

## Introduction

Photosynthesis is a fundamental physiological process that trees perform over fluctuating daily and seasonal environmental conditions. In urban areas, which can be up to 12 °C warmer than surrounding rural environments due to the urban heat island effect [1], trees may benefit from or be challenged by warmer temperatures, and by interactions between warming, drought stress, and air pollution [2–4]. Alone, warming and other abiotic changes associated with urbanization have additive effects on plant growth that may be positive or negative [5–8]; these factors may also influence tree physiology via their effects on trophic interactions [for example, 9–12; reviews by 13–15]. An urban tree’s ability to respond to environmental change, including warming, is a critical factor in its ability to provide ecosystem services that range from shading streets to carbon sequestration [16–18].

However, urban trees often differ not only in terms of the conditions to which they are exposed, but also in terms of their genotypes. For example, urban red maple (*Acer rubrum* L.) cultivars are likely to differ genetically and hence physiologically from wildtype trees. Naturally occurring trees (wildtype) are of genotypes likely to be adapted to regional climates [19, 20]. Wildtype trees do grow in urban areas including buffers, parks, and small forest fragments. However, most trees planted in cities are clonal cultivated varieties (cultivars) that are not of local provenance. A red maple cultivar may have been selected in the northeastern United States, propagated clonally, grown in a Pacific Northwest nursery, and then planted in a city in the southeastern United States. As a result, many urban trees have no adaptation to regional climates, or adaptation is contingent on whether the regional climate in which a cultivar is planted matches that of the region in which it originated. In addition, maple cultivars selected for particular leaf traits—such as color or size—may be more negatively affected by warmer regional environmental conditions [21] or temperature extremes and fluctuations that occur regularly in cities [1]. Such trees may not be locally adapted or have the chance to acclimate to regional climate, much less to an urban environment, before planting. This is a concern because many environmental conditions such as heat waves and droughts are already exacerbated in cities, and are predicted to increase further under climate change scenarios [22]. Sustainable urban forests and ecosystem services depend on understanding the trees selected for urban planting, including photosynthetic variation within a species and among horticultural varieties.

Our knowledge of the interacting physiological and ecological processes that govern photosynthesis and long-term tree condition and growth in cities is currently limited. While trees in warming experiments, particularly temperate forest species, generally respond positively to increases in temperature [3, 23–26, but see 4, 27], tree responses in cities are inconsistent. Depending on species, warmer urban trees have been shown to have lower rates of photosynthesis relative to rural trees or to trees in cooler urban areas [28, 29], higher rates of photosynthesis relative to trees in cooler urban areas [30], and no difference in rates of photosynthesis relative to rural trees [31]. Many studies have focused on photosynthetic variation among trees in natural environments [reviewed by 32] but much less is known regarding rates of photosynthesis and stomatal conductance for urban trees or cultivars. Our study is timely because there is little information on how photosynthesis varies due to warming relative to selection for cultivars with particular leaf traits or other genotypic or phenotypic differences.

We focus here on red maple. Wildtype red maple (*Acer rubrum* L.) is a ubiquitous component of early successional forests in the eastern United States [33, 34], and red maple cultivars and hybrid varieties are among the most commonly planted species of tree in eastern and Midwestern cities, a trend that is increasing [33, 35, 36]. However, there are over 40 different red maple hybrids and cultivars [37] many more genotypes, and little published data on how these different trees respond to urban conditions. To our knowledge, this study is the first to characterize photosynthesis, stomatal conductance, and water use efficiency in planted cultivars relative to wildtype trees (of any species), and to address how urban conditions influence photosynthetic variation.

**Fig 1:**
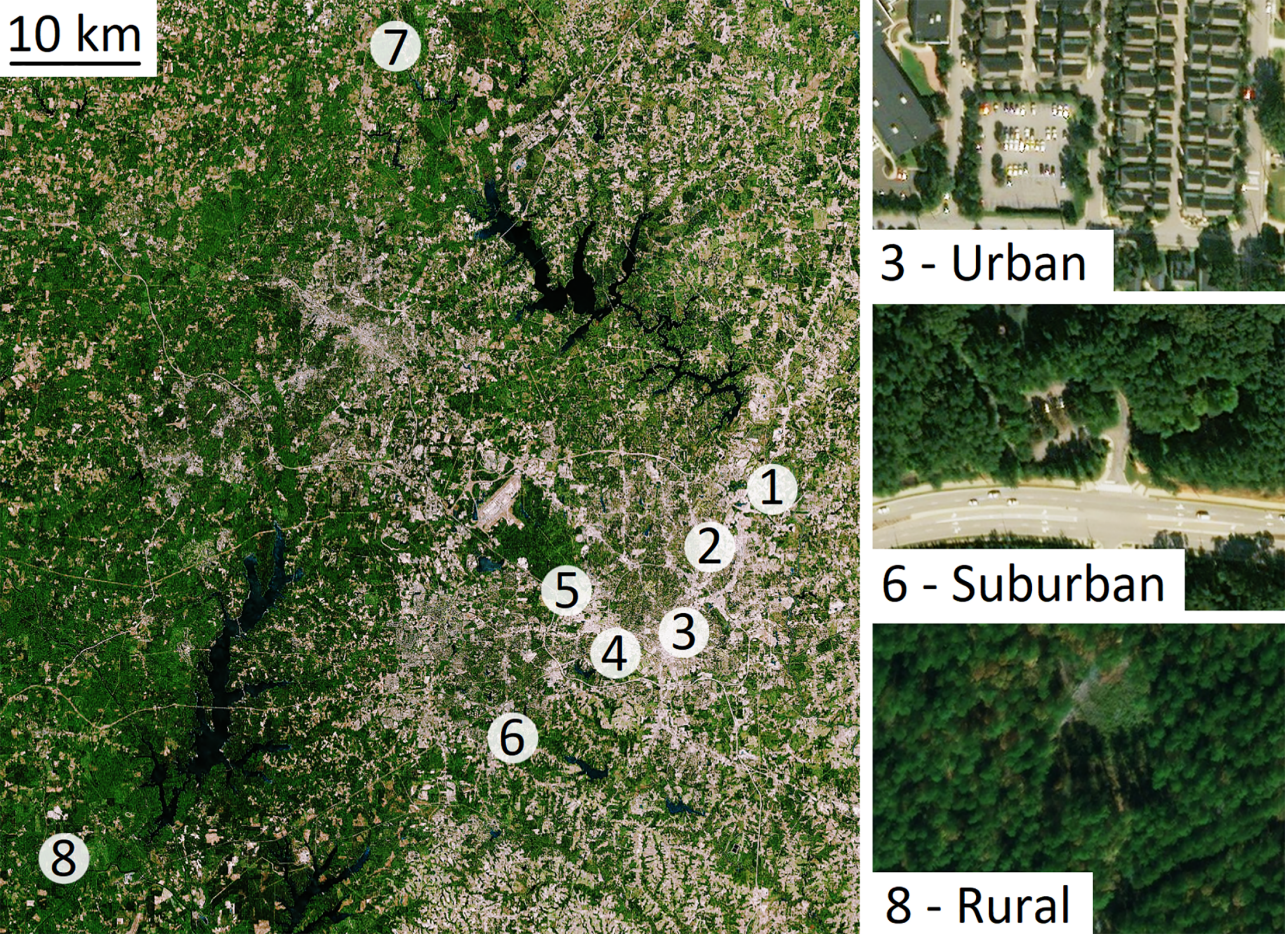
Map showing site locations for Experiment 1 in 2016. Sites 1–4 are urban locations in Raleigh, North Carolina (McGuire Dr., Falls of Neuse Rd., Harp St., and Varsity Dr., respectively), sites 5–6 are suburban locations (Carl Alwin Schenck Memorial Forest and Hemlock Bluffs State Nature Preserve, respectively), and sites 7–8 are rural locations (G.W. Hill Demonstration Forest and White Pines Nature Preserve, respectively). Side panels show detailed examples of an urban site (upper panel: Harp. St, site 3), a suburban site (middle panel: Hemlock Bluffs State Nature Preserve, site 6), and a rural site (lower panel: White Pines Nature Preserve, site 8). Dates of gas exchange measurements are given in Table 1. The images in Fig 1 were obtained from the USGS LandsatLook Viewer (images accessed April 18, 2018; https://landsatlook.usgs.gov/). The images have been annotated with identifying information.

**Fig 2:**
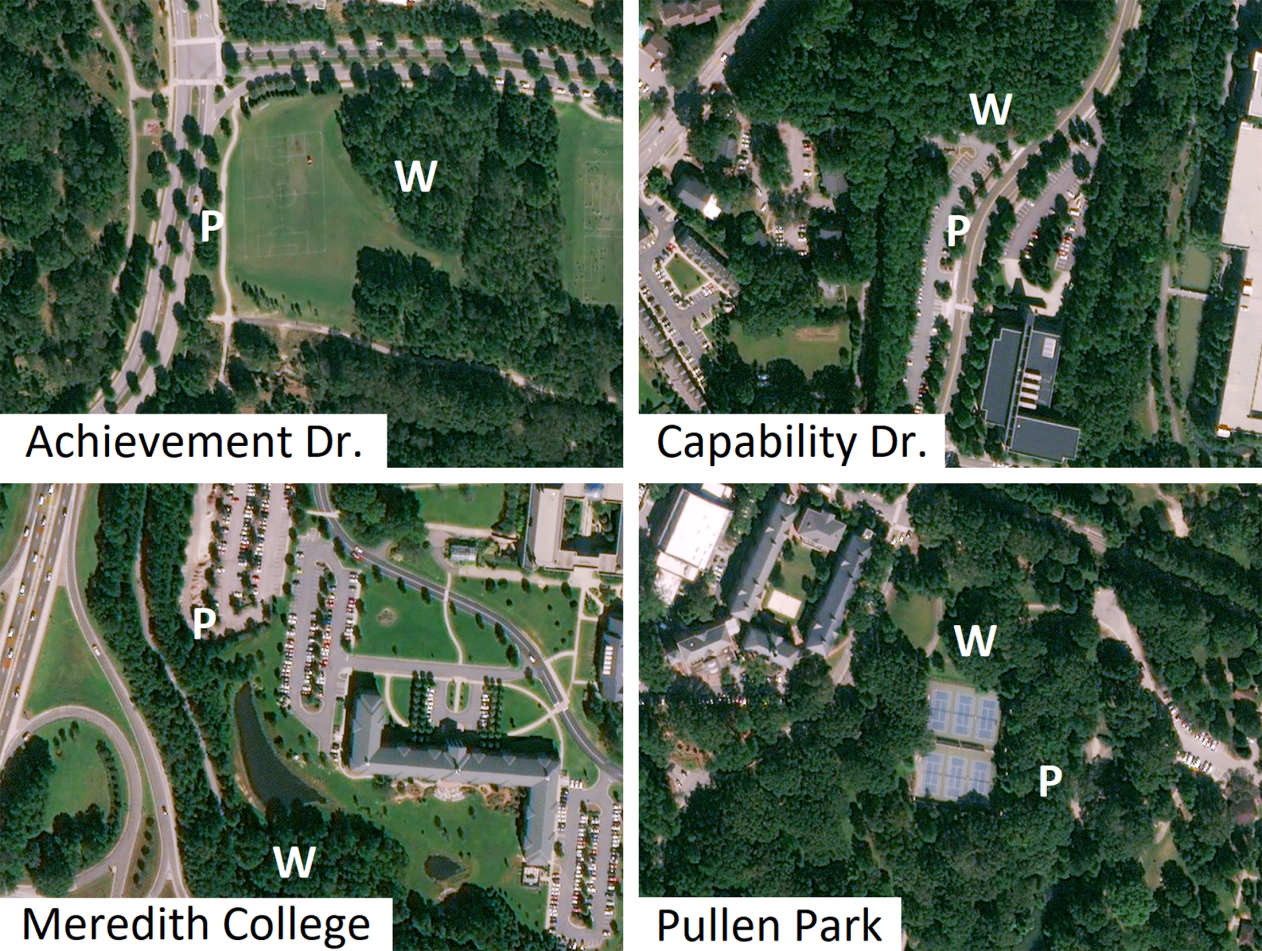
Map showing site locations in Raleigh, North Carolina for Experiment 2 in 2017. At each site, “P” indicates the area where gas exchange measurements on planted cultivars occurred, and “W” indicates an area where gas exchange measurements on wildtype trees occurred. Dates of gas exchange measurements are given in Table 1. The images in Fig 2 were obtained from the USGS LandsatLook Viewer (images accessed April 18, 2018; https://landsatlook.usgs.gov/). The images have been annotated with identifying information.

In this study, we measured gas exchange of trees in and around Raleigh, North Carolina, in the southeastern United States, for two years. We performed two experiments: First, we repeatedly measured leaf-level gas exchange and performed short-term temperature curves on planted cultivars at urban sites and wildtype trees at suburban and rural sites (Fig 1). In the second year, we compared planted cultivars and wildtype trees at urban sites (Fig 2). Leaf-level photosynthesis and stomatal conductance were measured at a standard temperature during the growing season, and we performed temperature curves to assess short-term responses to increasing temperatures. We asked the following questions in Experiment 1: (1) Do photosynthesis, stomatal conductance, and water use efficiency differ among urban, suburban, and rural sites during the growing season? and (2) Do rates of photosynthesis and stomatal conductance differ among urban, suburban, and rural sites during rapid, short-term changes in temperature? In Experiment 2 we asked: (3) Do photosynthesis, stomatal conductance, and water use efficiency differ between urban planted cultivars and urban wildtype trees?.

## Materials and methods

### Study area and species

Photosynthesis and stomatal conductance were measured in and around Raleigh, North Carolina, U.S.A. (35.772096 °N 78.638614 °W). Raleigh has a population of approximately 451,000 and a humid subtropical climate, with an average long-term January temperature of 4.2 °C and July temperature of 25.9 °C, and average annual precipitation of 117 cm (State Climate Office of North Carolina; www.climate.ncsu.edu; data accessed October 26, 2016). Raleigh is located in the Piedmont region of North Carolina, where red maple has increased as a component of immature secondary forests (Christiansen 1977). In its range throughout the eastern United States, red maple is a medium-sized tree with rapid growth and is tolerant of a range of environmental conditions. Its average lifespan is 80–150 years [34]. Red maple comprises over 18% of the trees in Raleigh’s urban street tree inventory [10].

#### Experiment 1

In 2016, planted trees (N = 4, mean diameter 29.2 ± 2.9 cm) were studied at four urban sites and wildtype trees were studied at two suburban sites (N = 7, mean diameter 17.7 ± 2.0 cm) and two rural sites (N = 7, mean diameter 12.4 ± 2.0 cm). Site locations are shown in Fig 1. Urban planted trees were located in City of Raleigh right-of-ways and were planted from 2002–2005 (Google Earth Pro Version 7.3.1 Historical Imagery; data accessed March 27, 2018). Urban trees were single-trunked with relatively transparent crowns. Suburban wildtype trees were located at Hemlock Bluffs State Nature Preserve, in the Town of Cary, and the Carl Alwin Schenck Memorial Forest, owned by North Carolina State University. Rural trees were located at White Pines Nature Preserve, owned by the Triangle Nature Conservancy, and the G.W. Hill Demonstration Forest, owned by North Carolina State University (Table 1, Fig 1). Suburban and rural trees were frequently double- or multi-trunked, with less transparent crowns, relative to urban trees. Gas exchange measurements were performed from July-October 2016. Mean rates of photosynthesis and stomatal conductance at each site and date of measurement for 2–4 leaves each for 1–5 trees, for each type of site (urban, suburban, and rural), are shown in Table 1.

**Table 1:** Gas exchange measurements for each type of site and tree.

Location	Site	Tree	Date	N	Photosynthesis (μ mol s^-1^ m^-2^)	Stomatal Cond. (μ mol s^-1^ m^-2^)
Raleigh, McGuire Dr.	U	planted	8/24/2016	3	7.042 ± 0.311	0.000 ± 0.000
			9/30/2016	3	8.314 ± 0.152	0.102 ± 0.013
Raleigh, Falls of Neuse Rd.	U	planted	8/10/2016	3	7.052 ± 0.698	0.076 ± 0.018
			9/23/2016	3	7.263 ± 0.876	0.059 ± 0.008
Raleigh, Harp St.	U	planted	8/18/2016	4	12.453 ± 0.659	0.174 ± 0.022
			9/28/2016	3	12.259 ± 0.874	0.150 ± 0.016
Raleigh, Varsity Dr.	U	planted	8/24/2016	2	6.851 ± 1.113	0.066 ± 0.010
			9/26/2016	4	12.732 ± 1.821	0.136 ± 0.013
Schenck Memorial Forest	S	wildtype	7/28/2016	12	8.152 ± 0.295	0.095 ± 0.005
			8/30/2016	9	5.917 ± 0.285	0.055 ± 0.004
			10/4/2016	9	6.067 ± 0.749	0.068 ± 0.008
Hemlock Bluffs Nature Preserve	S	wildtype	8/16/2016	11	5.991 ± 0.305	0.075 ± 0.006
			9/13/2016	12	5.137 ± 0.281	0.079 ± 0.012
			10/11/2016	9	4.720 ± 0.309	0.057 ± 0.005
White Pines Nature Preserve	R	wildtype	7/29/2016	12	4.993 ± 0.340	0.047 ± 0.004
			8/31/2016	8	2.855 ± 0.517	0.043 ± 0.008
			10/6/2016	6	4.503 ± 0.350	0.049 ± 0.002
Hill Demonstration Forest	R	wildtype	8/23/2016	9	5.365 ± 0.275	0.045 ± 0.003
			9/16/2016	6	5.901 ± 0.553	0.503 ± 0.008
			10/13/2016	6	7.382 ± 0.403	0.074 ± 0.006
Raleigh, Capability Dr. (NCSU)	U	planted	4/17/2017	13	10.750 ± 0.476	0.135 ± 0.008
			6/22/2017	12	12.500 ± 0.607	0.149 ± 0.011
			8/28/2017	12	6.831 ± 1.274	0.065 ± 0.014
	U	wildtype	4/17/2017	12	7.336 ± 0.674	0.076 ± 0.009
			6/22/2017	12	8.133 ± 0.448	0.111 ± 0.004
			8/28/2017	12	3.237 ± 0.836	0.037 ± 0.007
Raleigh, Achievement Dr. (NCSU)	U	planted	4/20/2017	15	10.592 ± 0.564	0.175 ± 0.014
			6/24/2017	12	10.284 ± 0.603	0.096 ±0.007
			8/23/2017	12	5.586 ± 0.750	0.072 ± 0.010
	U	wildtype	4/20/2017	15	8.402 ± 0.532	0.111 ± 0.011
			6/24/2017	12	11.018 ± 0.394	0.131 ±0.006
			8/23/2017	12	2.367 ± 0.305	0.028 ± 0.002
Raleigh, Pullen Park	U	planted	4/20/2017	12	7.117 ± 0.525	0.080 ± 0.006
			6/19/2017	12	7.737 ± 0.356	0.094 ± 0.006
			8/30/2017	12	4.771 ± 0.603	0.041 ± 0.006
	U	wildtype	4/20/2017	3	6.073 ± 1.107	0.061 ± 0.013
			6/18/2017	6	8.140 ± 0.426	0.093 ± 0.009
			8/30/2017	6	3.194 ± 0.772	0.026 ± 0.006
Raleigh, Meredith College	U	planted	4/21/2017	12	9.636 ± 0.621	0.106 ± 0.005
			6/23/2017	12	6.835 ± 0.536	0.081 ± 0.008
			8/22/2017	12	2.671 ± 0.675	0.032 ± 0.005
	U	wildtype	4/21/2017	12	6.872 ± 0.485	0.083 ± 0.008
			6/23/2017	12	6.991 ± 0.908	0.094 ± 0.010
			8/22/2017	12	5.016 ± 0.375	0.057 ± 0.005

Gas exchange measurements at each site are identified by type of site (U: urban, S: suburban, R: rural) in Experiment 1 in 2016 and type of tree (planted or wildtype) in Experiment 2 in 2017, for each sampling date. N = the total number of measurements. Photosynthesis and stomatal conductance (mean ± standard error) were measured for 2–4 leaves each from 1–5 trees.

#### Experiment 2

In 2017, planted trees (N = 16, diameter 22.1 ± 1.4 cm) and wildtype trees (N = 15 in total, diameter = 19.5 ± 3.5 cm) were studied at four urban sites (Table 1, Fig 2). Site selection was based on tree accessibility and the close proximity of wildtype trees and planted cultivars. At three sites (Meredith College, Achievement Dr., and Capability Dr.), wildtype trees grew at the edge of forest fragments and planted cultivars grew along adjacent parking areas less than 200 m away. These sites included a parking area at Meredith College with an adjacent greenway, and two parking areas at North Carolina State University (Achievement Dr., and Capability Dr.) with adjacent forest fragments. At the fourth site, a public park (Pullen Park), wildtype and planted cultivars grew together in an open area surrounding a tennis court. Trees at the first three sites were planted between 2002–2004 and trees at Pullen Park were planted prior to 1993 (Google Earth Pro Version 7.3.1 Historical Imagery; data accessed March 27, 2018). Planted cultivars were identified by the presence of a grafting scar at the base of the tree. Cultivars of red maple are commonly produced by bud grafting (Walters and Yawney 1990), which leaves a visible scar at the base of the tree. Gas exchange measurements were performed from April-September 2017. Mean rates of photosynthesis and stomatal conductance at each site and date of measurement for 3–4 leaves each for 2–5 trees, for trees of each type, are shown in Table 1.

#### Gas exchange measurements

Photosynthesis and stomatal conductance were measured using an LI-6400XT Portable Photosynthesis System with a leaf chamber fluorometer (LI-COR Biosciences Inc., Lincoln, Nebraska, U.S.A.). To minimize differences in light exposure, wildtype trees at the suburban and rural sites in 2016 and wildtype trees in urban forest fragments in 2017 were selected from the canopy edge and sun leaves on exterior branches 2–5 m above the ground were selected for *in situ* measurements. All planted urban trees received full sun. Gas exchange conditions followed [29]: 30 °C block temperature, 400 μmol mol^-1^ CO_2_, 1200 μmol m^-2^ s^-1^ photosynthetically active radiation, and vapor pressure deficit between 1–2 kPa. Standard measurement conditions, rather than ambient conditions, were used to control for variation in abiotic and biotic factors such as clouds and mid-day depression in photosynthesis, during the day of measurement and among measurement days. Following leaf stabilization in the LI-6400XT chamber, photosynthesis and stomatal conductance were recorded at 15 second intervals for 3 minutes. Instantaneous water use efficiency was calculated by dividing the rate of photosynthesis by the rate of stomatal conductance. Different leaves were selected on each sampling date and measurements were made between the hours of 10:00–16:00.

Temperature curves were performed following leaf measurement at standard gas exchange conditions. The LI-6400XT block temperature was lowered to 26 °C and increased in 4-degree increments until 38 °C. Vapor pressure deficit was not controlled during temperature curves but photosynthetically active radiation and CO_2_ concentration remained at the settings described above. After leaves stabilized at each temperature increment, photosynthesis and stomatal conductance were recorded at 15 second intervals for 3 minutes.

Ambient air temperature at the time of gas exchange measurements was recorded using iButton thermochron DS1921G remote temperature loggers (Dallas Semiconductor, Dallas, Texas, U.S.A.). At each site, iButtons were placed approximately 4.5 m above the ground within the tree canopy, mounted on the underside of a lateral branch, according to [10]. Temperature was recorded every hour. In 2016, temperature data from Weather Underground personal weather stations, recorded at 10-minute intervals, was used to fill gaps due to incomplete coverage (https://www.weatherunderground.com; data accessed November 8, 2016). To insure data quality, Weather Underground and iButton data were regressed against ambient temperature readings obtained by the LI-6400XT during gas exchange measurements and were found to be tightly correlated (data not shown). All temperature data will be available along with gas exchange data in the KNB data repository.

#### Statistical analyses

Statistical analyses were performed in R version 3.3.0 [38]. General linear mixed models were used to assess photosynthesis, stomatal conductance, and instantaneous water use efficiency (the ratio of photosynthesis to stomatal conductance) in Experiments 1 and 2 using the “lme4” package [39] with Kenward-Roger approximation of *F*-statistics [40, 41], and planned post hoc comparisons of selected treatments using the “lsmeans” package [42]. Models for photosynthesis, stomatal conductance, and water use efficiency were performed separately.

In Experiment 1, main effects included ambient air temperature and site type (urban, suburban, and rural), with tree diameter as a covariate. Site type × air temperature interactions were included to assess the influence of air temperature (which is frequently correlated with urban impervious surface; e.g. [10–12, 43] on gas exchange at different types of sites. Random effects included tree identity, to account for diurnal change in ambient conditions on sampling days, and measurement date, to account for repeated measures. General linear mixed models were also used to assess photosynthesis and stomatal conductance during temperature curves. Main effects included temperature (which was increased experimentally in 4-degree increments from 26–38 °C), site type (urban, suburban, and rural) and the temperature × site type interaction. Random effects included tree identity.

In Experiment 2, main effects included site (Achievement Dr., Capability Dr., Meredith College, and Pullen Park), tree type (planted and wildtype), and season (April, June, and August), with tree diameter, and ambient air temperature as covariates. Site × tree type, site × season, tree type × season, tree type × air temperature, and site × tree type × season interactions were performed. Random effects included tree identity.

## Results

**Fig 3:**
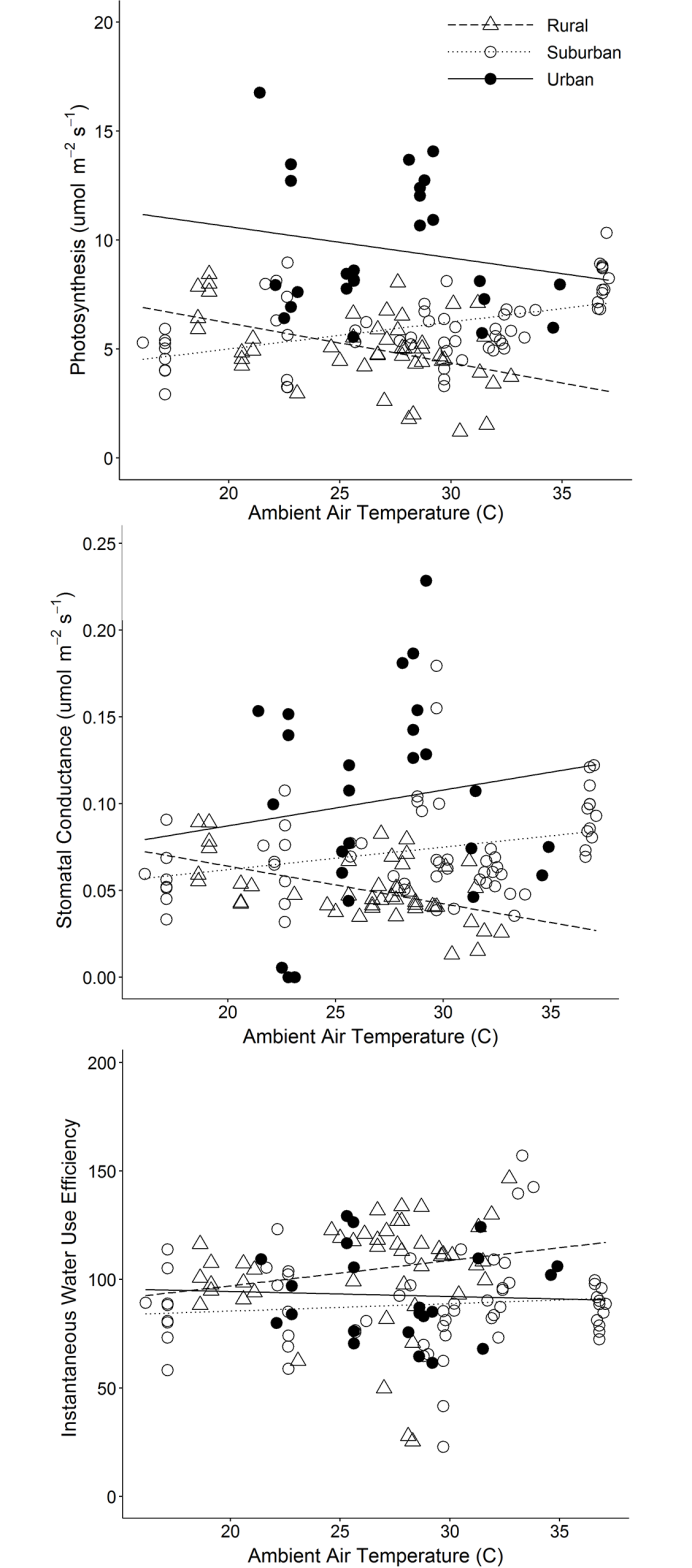
Mean rates of gas exchange during Experiment 1 in 2016. Photosynthesis (upper panel), stomatal conductance (middle panel), and instantaneous water use efficiency (the ratio of photosynthesis to stomatal conductance; lower panel) are shown relative to ambient air temperature. Closed circles show urban sites, open circles show suburban sites, and open triangles show rural sites. Lines represent model predictions (Table 2) at urban (solid line), suburban (dotted line) and rural (dashed line) sites, after accounting for effects of tree diameter.

In Experiment 1, site type significantly influenced rates of photosynthesis, but not stomatal conductance or water use efficiency, over the growing season (Question 1). Urban trees (all planted cultivars) had higher rates of photosynthesis than did suburban or rural trees (all wildtype) (Table 2, Fig 3). Neither tree diameter nor ambient air temperature influenced photosynthesis, stomatal conductance, or water use efficiency at the leaf level, but a significant site type × air temperature interaction occurred for photosynthesis and stomatal conductance (Table 2). Photosynthesis decreased as ambient air temperature increased at urban and rural sites and increased with air temperature at suburban sites (Fig 3). Stomatal conductance increased with air temperature at urban and suburban sites and decreased with air temperature at rural sites (Fig 3). Water use efficiency was not significantly influenced by site type, air temperature, or site type × air temperature.

**Table 2:** General linear mixed models test the effects of site type, tree diameter, and air temperature on gas exchange.

Variable	Photosynthesis			Stomatal Cond.			Water Use Efficiency		
	*F*	*df*	*P*	*F*	*df*	*P*	*F*	*df*	*P*
Site type	11.254	2, 127	**≤ 0.001**	1.821	2, 127	0.166	0.064	2, 124	0.938
Tree diameter	0.407	1, 127	0.524	0.694	1, 127	0.407	3.014	1, 124	0.085
Air temperature	2.639	1, 127	0.107	0.299	1, 127	0.586	0.64	1, 124	0.425
Site type × Air temperature	9.801	2, 127	**≤ 0.001**	3.682	2, 127	**0.028**	0.553	2, 124	0.577

Explanatory variables include site type (rural, suburban, urban), tree diameter, ambient air temperature, and site type × air temperature. Response variables include photosynthesis (μ mol s^-1^ m^-2^), stomatal conductance (μ mol s^-1^ m^-2^), and instantaneous water use efficiency (the ratio of photosynthesis to stomatal conductance) in Experiment 1 in 2016. Random effects include tree identity and measurement date. Bold P-values indicate significant effects.

**Fig 4:**
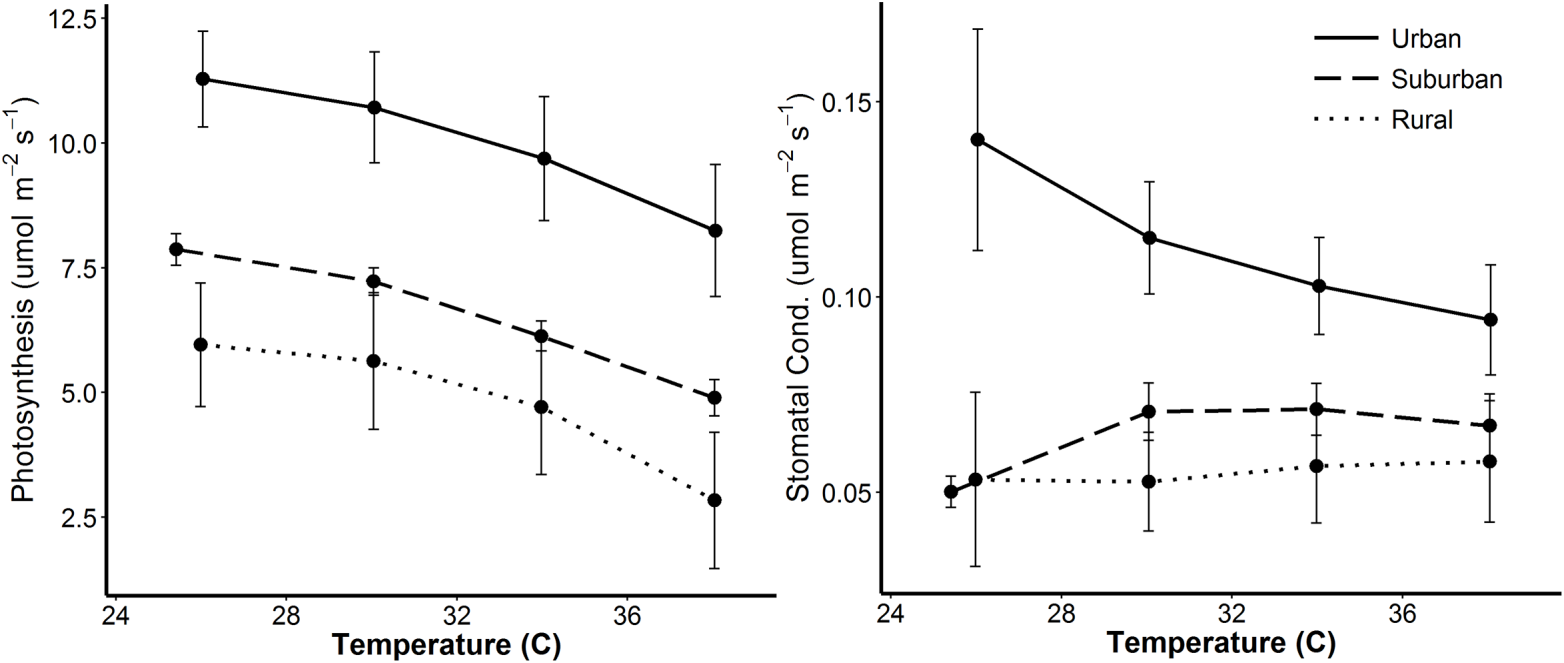
Experimental manipulation of temperature in Experiment 1 in 2016. Photosynthesis (left panel) and stomatal conductance (right panel) of red maples are shown. Points show means of 4–8 measurements per urban (solid line), suburban (dashed line) and rural (dotted line) sites. Error bars indicate ± standard error.

Site type also influenced rates of photosynthesis and stomatal conductance when temperature was manipulated in the field (Question 2). During temperature curves, photosynthesis was correlated with site type (*F*_2,13_ = 5.636, *P* = 0.017) and was higher at urban relative to suburban or rural sites (Fig 4). Photosynthesis was also correlated with temperature and declined as temperatures increased (*F*_3,39_ = 46.194, *P* ≤ 0.001; Fig 4). Stomatal conductance was correlated with site type (*F*_2,13_ = 5.204, *P* = 0.023) and was higher at urban relative to suburban or rural sites (Fig 4). There was no significant temperature × site type interaction.

**Fig 5:**
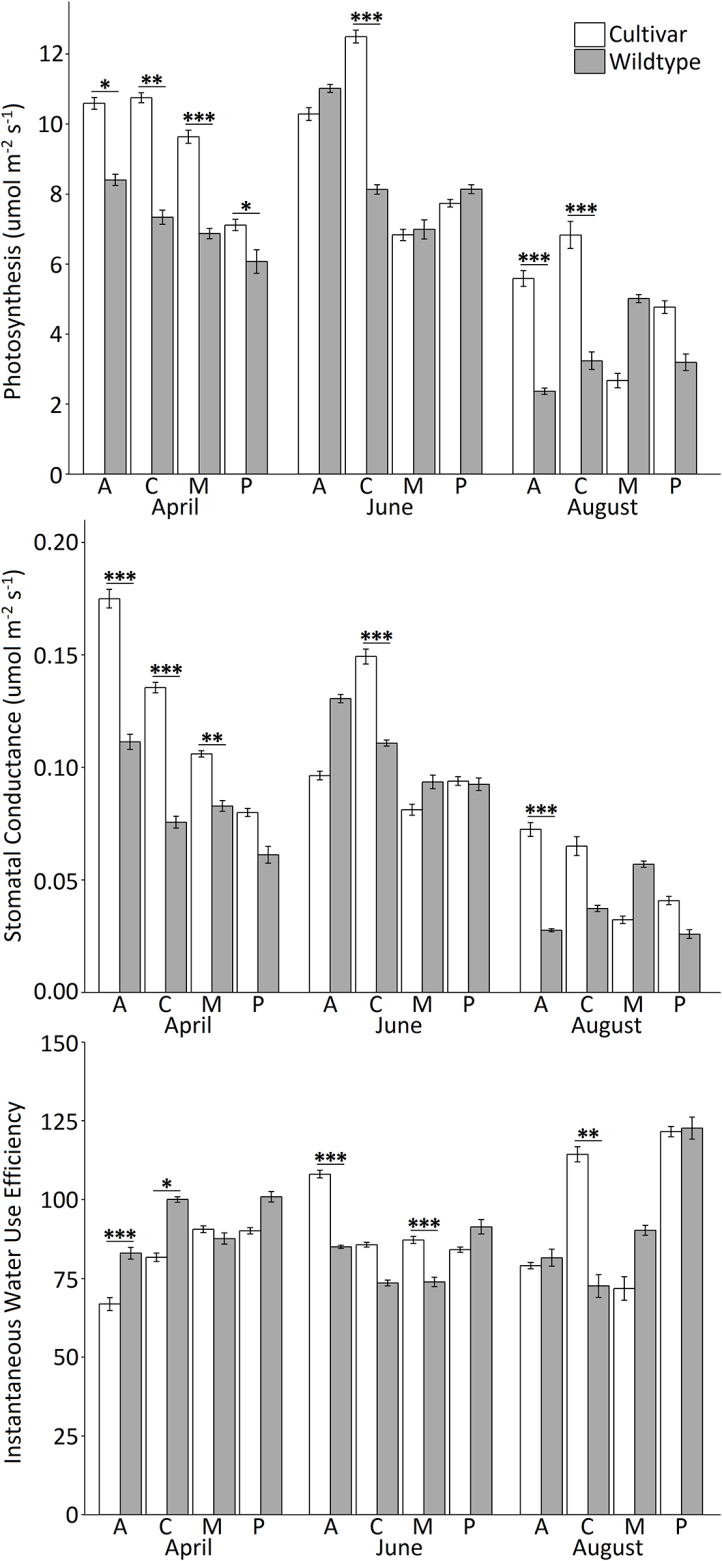
Mean rates of gas exchange during Experiment 2 in 2017. Photosynthesis (upper panel), stomatal conductance (middle panel), and instantaneous water use efficiency (the ratio of photosynthesis to stomatal conductance; lower panel) are shown. White bars show planted trees and grey bars show wildtype trees, at each site (A: Achievenemt Dr., C: Capability Dr., M: Meredith College, P: Pullen Park) in each season (April, June, and August). Error bars show ± standard error. Horizontal bars show significant contrasts according to post hoc pairwise comparisons, with * indicating p ≤ 0.05, ** indicating p ≤ 0.001, and *** indicating p ≤ 0.0001.

**Fig 6:**
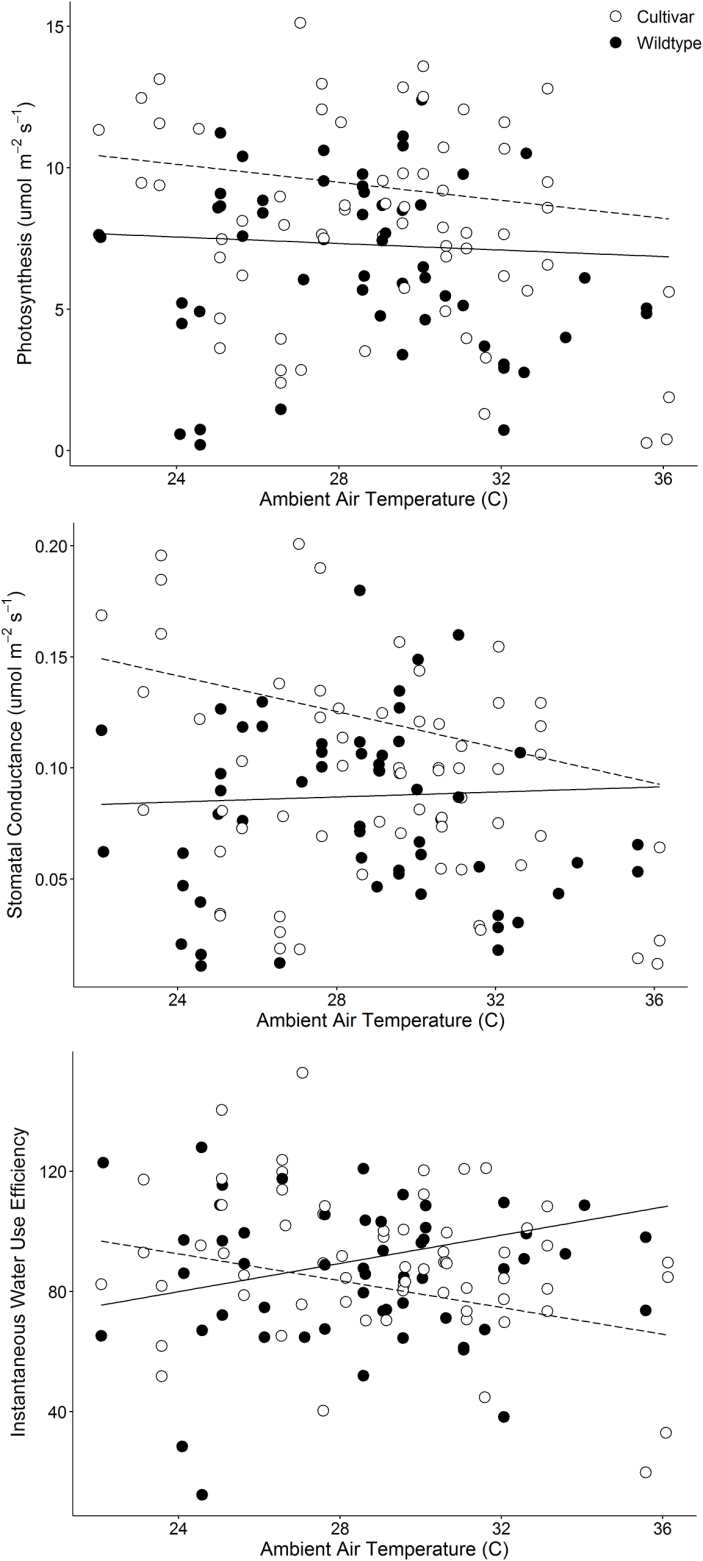
Mean rates of gas exchange during Experiment 2 in 2017. Photosynthesis (upper panel), stomatal conductance (middle panel) and instantaneous water use efficiency (the ratio of photosynthesis to stomatal conductance; lower panel) are shown. Open circles represent planted cultivars and filled circles represent wildtype trees, combined among sites. Lines represent model predictions for planted cultivars (dashed line) and wildtype trees (solid line), after accounting for effects of tree diameter and site.

Experiment 2 was designed to determine whether higher rates of photosynthesis in urban trees—observed in Experiment 1—were related to site type (urban, suburban, or rural) or to tree type (planted cultivar or wildtype). In Experiment 2, site, tree type, and season had significant effects on photosynthesis, stomatal conductance, and water use efficiency (Question 3). Overall, planted cultivars had higher rates of photosynthesis and stomatal conductance, but inconsistent differences in water use efficiency for planted cultivars versus wildtype trees occurred depending on site and season (Table 3, Figs 5 and 6). Tree diameter did not significantly influence gas exchange. Ambient air temperature significantly influenced rates of photosynthesis and stomatal conductance. Significant site × tree type, site × season, tree type × season, and site × tree type × season interactions occurred for photosynthesis (Table 3). Planted trees had higher rates of photosynthesis relative to wildtype trees at all sites in April, at Capability Dr. in June, and at Achievement Dr. and Capability Dr. in August (Fig 5). Wildtype trees did not have higher rates of photosynthesis at any time, however, there were no significant differences in photosynthesis between planted cultivars and wildtype trees at Achievement Dr., Meredith College, or Pullen Park in June, or between planted and wildtype trees at Meredith College or Pullen Park in August (Fig 5). Significant site × season, tree type × season, tree type × air temperature, and site × tree type × season interactions occurred for stomatal conductance and water use efficiency (Table 3). Planted cultivars had higher rates of stomatal conductance at Achievement Dr., Capability Dr., and Meredith College in April, at Capability Dr. in June, and at Achievement Dr. in August. Wildtype trees did not have higher rates of stomatal conductance at any time (Fig 5). Planted cultivars had higher water use efficiency at Achievement Dr. and Meredith College in June, and at Capability Dr. in August. Wildtype trees had higher water use efficiency at Achievement Dr. and Capability Dr. in April (Fig 5). Overall, mean rates of photosynthesis were higher for planted cultivars, relative to wildtype trees, as air temperatures increased, but stomatal conductance was generally lower in wildtype trees (Fig 6). Water use efficiency was negatively correlated with air temperature at all sites for planted cultivars, and positively correlated with air temperature at all sites for wildtype trees (Fig 6).

**Table 3:** General linear mixed models test the effects of site, tree type, season, tree diameter, and air temperature on gas exchange.

Variable	Photosynthesis			Stomatal Conductance			Water Use Efficiency		
	*F*	*df*	*P*	*F*	*df*	*P*	*F*	*df*	*P*
Site	8.55	3, 101	**≤ 0.001**	9.80	3, 100	**≤ 0.001**	6.897	3, 105	**≤ 0.001**
Tree type	5.73	1, 2591	**0.017**	41.16	1, 2656	**≤ 0.001**	46.577	1, 2279	**≤ 0.001**
Season	2241.65	2, 2923	**≤ 0.001**	2533.41	2, 2914	**≤ 0.001**	43.794	2, 2940	**≤ 0.001**
Tree diameter	2.70	1, 97	0.103	0.89	1, 96	0.348	1.509	1, 99	0.222
Air temperature	11.78	1, 2840	**≤ 0.001**	0.21	1, 2971	**≤ 0.001**	0.032	1, 2457	0.859
Site × Tree type	3.43	3, 100	**0.020**	2.32	3, 99	0.080	1.419	3, 104	0.241
Site × Season	64.74	6, 2944	**≤ 0.001**	91.06	6, 2935	**≤ 0.001**	109.135	6, 2942	**≤ 0.001**
Tree type × Season	37.49	2, 2924	**≤ 0.001**	111.82	2, 2914	**≤ 0.001**	50.559	2, 2940	**≤ 0.001**
Tree type × Air temperature	2.57	1, 2860	0.109	32.35	1, 2955	**≤ 0.001**	46.453	1, 2499	**≤ 0.001**
Site × Tree type × Season	70.57	6, 2943	**≤ 0.001**	86.75	6, 2933	**≤ 0.001**	52.111	6, 2943	**≤ 0.001**

Explanatory variables include site (Achievement Dr., Capability Dr., Meredith College, Pullen Park), tree type (cultivar, wildtype), season (April, June, August), tree diameter, ambient air temperature, and appropriate interactions relative to the reference category (Achievement Dr., cultivar, April). Response variables include photosynthesis (μ mol s^-1^ m^-2^), stomatal conductance (μ mol s^-1^ m^-2^), and instantaneous water use efficiency (the ratio of photosynthesis to stomatal conductance) in Experiment 2 in 2017. Random effects include tree identity. Bold P-values indicate significant effects.

## Discussion

Urban trees provide a range of ecosystem services and social benefits which range from shading streets, improving air quality, and sequestering carbon [16, 44, 45]. Although these benefits motivate urban tree planting throughout the United States and around the world, we still know little about the physiological processes such as photosynthesis that shape long-term tree condition and growth in cities. Red maple is a popular urban planting choice in the eastern United States, but selecting for horticultural varieties based on appearance does not necessarily ensure long-term tree health and growth. Here, we show that urban planted cultivars reached higher maximum rates of photosynthesis and maintained higher rates of photosynthesis during temperature manipulation, relative to suburban and rural wildtype trees. However, after our first experiment, it was unclear whether higher rates of photosynthesis in urban trees were related to site type (urban, suburban, or rural) or to tree type (planted cultivar or wildtype). Warmer temperatures have been shown to stimulate photosynthesis in trees [25, 26, 30] and planted red maples could have experienced higher rates of photosynthesis and stomatal conductance due to the urban heat island effect [1], or, higher rates of photosynthesis and stomatal conductance could have occurred in urban planted cultivars due to selection for genotypes with particular leaf traits. Our results support the latter possibility. Ultimately, differences in gas exchange between urban planted cultivars and urban wildtype trees led to differences in water use efficiency that may influence long-term tree growth and condition.

In Experiment 1, we saw that red maples planted along urban streets had higher rates of photosynthesis and stomatal conductance than did wildtype trees in suburban or rural forests (Question 1), independent of tree diameter, date of measurement, or site type × air temperature interactions (Fig 3). While urban sites had higher rates of photosynthesis overall, these declined with increasing temperature, converging upon rates of photosynthesis at suburban sites. It is unclear why photosynthesis had a slight positive relationship with temperature in suburban sites, but not in rural or urban sites, but these effects may have been due to different site conditions or weather conditions preceding measurements. However, it is important to note that all gas exchange measurements occurred at a standard leaf temperature of 30 °C and vapor pressure deficit between 1–2 kPa, indicating that these effects were due to different site conditions rather than different leaf temperatures at the time of measurement (Fig 3).

When leaf temperatures at each site were experimentally manipulated (Question 2), causing overall declines in photosynthesis at all sites as temperatures were raised, urban trees still maintained higher rates of photosynthesis and stomatal conductance than did suburban or rural trees (Fig 4), indicating that urban trees have a greater temperature range for photosynthesis. Despite these temperature curves being on a short time scale, they are relevant both physiologically and ecologically. Leaf temperatures and rates of photosynthesis can change dramatically in response to short-term changes in sun exposure such as sunflecks [46], which also have significant microclimate effects for arthropods [47, 48].

To clarify whether variation in rates of photosynthesis and stomatal conductance were due to site type or tree type, we then compared urban planted cultivars to urban wildtype red maples in adjacent forest fragments in Experiment 2. Planted trees had higher maximum rates of photosynthesis than did wildtype trees (Table 2), however, these high rates occurred primarily in April; planted cultivars and wildtype trees were not significantly different at three of four sites in June and two of four sites in August (Fig 5). Overall, planted cultivars also had higher stomatal conductance than wildtype trees, and this both enabled higher rates of photosynthesis by cultivars and led, at times, to higher water use efficiency by wildtype trees (Fig 5). Water use efficiency was influenced by both season and site (Table 3, Fig 5), but overall, planted cultivars and wildtype trees demonstrated clear differences in response to ambient air temperatures. As temperature increased, water use efficiency declined in planted cultivars and increased in wildtype trees (Fig 6), indicating that higher photosynthesis in cultivars is partially contingent upon water availability.

Lower water use efficiency may contribute to water stress and to reductions in carbon storage over the long term. While we observed no indication that these planted urban red maples were affected by water stress in 2016 or 2017, drought stress does commonly occur in city trees due to greater amounts of impervious surface [43, 49]. If soil water becomes limiting or temperature too high, stomatal conductance will decline to prevent xylem embolism, and trees will experience a corresponding reduction in rates of photosynthesis [50, 51]. Although the planted cultivars we observed were able to maintain higher rates of stomatal conductance in 2017, such higher rates of conductance and lower water use efficiency may eventually make trees more prone to xylem embolism during high temperatures. At the same time, however, stomatal conductance contributes significantly to leaf cooling [46, 50]. Water usage and availability are therefore important factors to consider in urban tree planting strategies as warmer urban temperatures can have a large effect on evapotranspiration, tree growth, and long-term sustainability [16, 52, 53].

Environmental conditions such as higher urban CO_2_ concentrations [54–56], and habitat differences, such as greater amounts of urban impervious surface, may also have influenced our results for photosynthesis and stomatal conductance. We controlled for these factors during our study by measuring photosynthesis and stomatal conductance at a standard light level, temperature, and CO_2_ concentration within the LI-6400XT chamber, but it is likely that urban sites in Raleigh experienced a higher background concentration of CO_2_ relative to our suburban and rural sites, a pattern documented in urban-rural comparisons in Baltimore and New York City [54–56]. Higher CO_2_ concentrations may have facilitated higher rates of photosynthesis by urban trees if CO_2_ was limiting or if drought stress was mitigated by greater CO_2_ availability, which would reduce water loss via stomatal conductance. However, despite potential interactions between these environmental variables, site type (urban, suburban, or rural) and tree type (planted cultivar or wildtype) provided strong discriminatory power in our analyses. This is important knowledge for cities, because urban forest sustainability may be improved by planting wildtype trees in areas where a less uniform tree appearance is acceptable, such as buffer areas around city parks. While such trees may have lower maximum rates of photosynthesis, they may achieve similar long-term carbon storage by maintaining equivalent rates of photosynthesis relative to planted cultivars, through the middle of the growing season. Greater water use efficiency as air temperatures increase may also contribute to improved tree condition in more stressful urban environments or during droughts or heat waves. The particular value of our study is in demonstrating the potential to use site characteristics such as impervious surface cover [46] and tree type to inform appropriate tree planting locations for red maple.

Red maple has a wide geographic range and environmental tolerance, which contributes to its popularity as an urban street tree, but interactions between biotic and abiotic factors have only begun to be investigated in urban landscapes. A key concern for urban forest sustainability is whether planting thousands of clones from a single horticultural variety, rather than increasing genetic diversity through natural selection, makes urban tree populations more susceptible insect pests and pathogens [57, 58]. Clear relationships have been found between temperature, impervious surface, and drought, and pest abundance and poor condition of red maples within cities [9, 43]. Although we included ambient air temperature in our models of gas exchange, in the future it will be important to investigate the relationship between tree type, habitat (i.e. differences in impervious surface), and tree condition. For example, [59] found that urban red maples had a greater abundance of scale insects than trees in rural forests, but it is unclear whether arthropods interact differently with planted versus wildtype trees in cities, or how impervious surface may affect the condition of planted versus wildtype trees in cities. Moreover, we could not identify specific cultivars in our study because that information is not included in tree inventories for Raleigh or many other cities (S.D. Frank unpublished data). If more cities included cultivar or hybrid identity in their lists of approved urban trees and in their urban tree inventories in the future, it would be possible to perform long-term evaluations of the susceptibility, condition, and performance of different horticultural varieties in diverse urban locations and in cities across multiple climate zones. Ultimately, we may improve integrated pest management and reduce costs of frequently removing and replanting trees in poor condition by better understanding the physiological and ecological differences between wildtype trees and planted cultivars.

Our results demonstrate that differences in genetic background are important to consider in urban tree planting and in the use of cultivars in manipulative experiments. Overall, wildtype trees should be considered for some urban locations due to the direct and indirect benefits of higher water use efficiency. For example, warming may directly enhance photosynthesis, but it may indirectly reduce tree growth and condition by exacerbating abiotic and biotic stresses such as drought and herbivory. Spatial models and techniques have been developed to assist city planners in selecting optimal tree species and planting locations [18, 49, 60], but to insure long-term sustainability of urban forests, we must continue to develop an understanding of urban tree physiology and ecology following planting, and integrate data on site type and cultivar identity into our management techniques. This will allow cities to maximize urban ecosystem services while minimizing the management costs that result from poor urban tree performance [16–18]. Accounting for differences between wildtype and planted trees under fluctuating short- and long-term environmental conditions is an example of how urban ecological research may contribute to this goal while additionally complementing experimental and modeling studies of tree responses to environmental change.
